# Relating biodiversity with health disparities of human population: An ecological study across the United States

**DOI:** 10.1016/j.onehlt.2023.100548

**Published:** 2023-04-23

**Authors:** Ying Chen, Peng Zhao, Qiaochu Xu, Bingjie Qu, Dan Li, Sarah Clement, Li Li

**Affiliations:** a Wisdom Lake Academy of Pharmacy, Xi'an Jiaotong-Liverpool University, Suzhou 215123, China; b Department of Health and Environmental Sciences, Xi'an Jiaotong-Liverpool University, Suzhou 215123, China; c Institute of Systems, Molecular & Integrative Biology, Faculty of Health & Life Sciences, University of Liverpool, UK; d Fenner School of Environment and Society, Australian National University, Canberra, Australia 2601. Department of Geography & Planning, School of Environmental Science, University of Liverpool, UK

**Keywords:** Avian diversity, Kunming-Montreal Global Biodiversity Framework, Sustainable development goals, One health, Life expectancy, Human–biodiversity interaction

## Abstract

Understanding biodiversity's contributions to human health is the first step toward fostering synergies between biodiversity conservation and health promotion - two major targets of UN's Sustainable Development Goals. The One Health approach acknowledges the health of people and biodiversity are interconnected and facing common threats. In this study, we aimed to unveil the geographical association between avian biodiversity and population health across the US. In this ecological study, we combined citizen science bird data from eBird, population health data from the Institute for Health Metrics and Evaluation, and county-level statistics of population characteristics, including socio-economics, healthcare service etc. Multivariate linear regression analyses were performed between bird biodiversity (measured by rarefied species richness of birds), key indicators of general public health (e.g., cause-specific mortality rate), and socio-economic health determinants of 2751 US counties. We found that a higher number of bird species was significantly associated with longer life expectancy after confounding adjustment (regression coefficient (95% CIs), 0·005 (0·003, 0·008)). Bird species richness calculated using the rarefied method consistently accounted for variance in age-specific mortality risks in both very young and old age groups (R^2^ from 2% to 4%). Rarefied species richness of birds was negatively correlated with the majority of cause-specific deaths (12 out of 21 mutually exclusive causes of death), indicating a general synergy effect between biodiversity and human health. The associations with the top causes of deaths were regarded as highly significant, with considerable effect sizes, for example, for cardiovascular diseases (regression coefficient (95% CIs), −0·242 (−0·311, −0·174)). Our findings show human health is inseparable from the health of the shared environment and the well-being of all species. Bird species richness offers a valuable means to understand large-scale relationships between human health and the health of the environment. To enable equitable sharing of biodiversity's benefits to human health, more efforts should be made to understand two-way socio-ecological mechanism underlying human–biodiversity interactions.

## Highlights

• Higher bird diversity is positively associated with better health at the regional scale.
• Avian diversity benefits health, evident in varied cause-specific deaths.
• Human health is intricately linked to the health of the shared environment and the well-being of all species.
• Species richness of birds aids understanding of regional-level human-environment health links.

## 1 Introduction

Biodiversity is a public good that healthy natural systems provide to human societies [1]. Nature and biodiversity underpin planetary health, provision of valuable ecosystem services and act as the foundation for human well-being [2]. For human health in particular, biodiversity is considered to mark a crucial ‘boundary condition’ sustaining health-benefiting ecosystem functions [3]. An increasing body of literature has reported positive effects of biodiversity on mental health through mediating mechanisms including stress reduction and attention restoration [4]. Interestingly, biodiversity's beneficial effects on mental health may depend on human-perceived biodiversity instead of surveyed species in the surrounding area [5]. This finding leads to a critical question: to what extent does the real presence of biodiversity influence health of people living in the shared environment?

There are different schools of contemporary holistic thinking that relate human health to the health of the total environment. Aaron Antonovsky developed the interdisciplinary *salutogenesis* framework [6], in which health is a continual movement of creating coherence between the individual and his or her social and environmental resources [7]. Calvin Schwabe in 1964 elaborated the *One Medicine* concept, arguing that combating diseases, providing sufficient food, and ensuring quality environment should be addressed in a whole to promote human health [8]. In 2004, the *One Health* concept was first phrased by the Wildlife Conservation Society, and the holistic approach was rapidly acknowledged by intergovernmental institutes including the Food and Agricultural Organization (FAO), United Nation's Environmental Program (UNEP), and recently the Intergovernmental Science-Policy Platform on Biodiversity and Ecosystem Services (IPBES) [9,10]. The *One Health* approach considers the health of people, animals, plants and the ecosystem in which they coexist, which are interconnected and facing common threats [1,11]. Biodiversity, as a measure of genetic, species and ecosystem variability of all life forms of the earth, is a keystone component of the material foundations for physical health [12]. The absence of certain living organisms can indicate health-undermining environmental stressors, such as lichens for clean air [13]. However, the pathways of biodiversity's impacts on physical health are still contested. For example, ecological communities of high biodiversity can inhibit the spread of parasites via regulating populations of susceptible hosts, while there are also concerns regarding to what level biodiversity can increase the risk of people's exposure to zoonotic pathogens [14,15]. Generally speaking, empirical evidence remains scarce with regard to the impact of biodiversity, either positive or negative, on physical health [16]. Particularly, there has been little quantified evaluation regarding cumulative effects that can be measured by rigid public health metrics such as life expectancy, age-specific mortality risk, or cause-specific mortality rate [3].

From the perspective of nature conservation, the year 2022 marks the start of aspirations for transformative change [17]: The 15th Conference of the Parties (COP15) of the Convention on Biological Diversity (CBD) has adopted the Kunming-Montreal Global Biodiversity Framework, which is commited to increase the global cover of land protected area from 17% in CBD's Aichi Targets to 30% by the year 2030 [2]. Under the new conservation paradigm, the coexistence of human and wild flora and fauna will prevail. As such, conservation managers and public health professionals are striving to illustrate empirical biodiversity–health patterns, which can be used to inform conservation planning in human landscapes [18]. The new biodiversity framework also adopts a nexus approach, with the most recent work programme focused on interlinkages between biodiversity, health and other elements that matter for a good quality of life [19].

One of the most widely used biodiversity indicators is the species richness of birds [20]. Species of the taxon occupy nearly all terrestrial environments and are windows into biotic processes at all levels. Birds are plentiful, mostly diurnal, and behaviorally and morphologically conspicuous [21]. Most importantly, birds are sensitive to disturbances, often heralding key changes in environmental processes or ecosystem health. Species richness has been proven to be an effective biodiversity metric in environment–health studies of large scales [22]. In this study, we utilized the best available citizen science bird database to calculate rarefied species richness of birds, which accounted for variations in sample efforts. We aimed to explore the relationship between biodiversity and important health measurements. We chose the United States (US) as the case study, because the US has a robust resource recorded data on both human health and bird diversity, and a high county-level variations in both bird species richness and human health proxies, as well as its large geographical scale, with data available for a significant proportion of the continent. We tested two hypotheses: First, across the US, we can detect associations between biodiversity and measurements of overall health (including life expectancy at birth, age-specific mortality risk and cause-specific mortality rate). Second, according to the One Health concept, biodiversity can mitigate health risks among different age groups, as well as mortality from a high variety of causes. Besides testing these two hypotheses, we were also interested to know the relative importance of biodiversity to life expectancy in comparison with other influential factors such as demographics, socio-economic characteristics, level of healthcare service, residential environment and geographical location.

## 2 Material and methods

### 2.1 Setting and design

This was an ecological study across the US where the sample unit was at the county (or county equivalent) level, with two areas of data: 1) rarefied species richness of birds as an indicator of county-level biodiversity, and 2) county-level statistics of population health. In addition, characteristics of the population, socio-economics, healthcare service, residential environment and geographical location in the US by county were also included for potential confounding adjustment.

### 2.2 Rarefied species richness of birds

Data on richness of bird species was obtained from the eBird database [23]. It is a program lunched by the Cornell Lab of Ornithology and the National Audubon Society in 2002. It engages a vast network of human observers (citizen scientists) to report bird observations using standard protocols, with its mission to transform the global birding community's passion for birds into a powerful resource for research, conservation, and education [[24], [25], [26]]. The eBird database was found to be comparable with the more comprehensive North American Breeding Bird Survey with regard to producing consistent multi-year abundance trends for bird populations at the national and regional scales [27].

In the current study, we extracted the data from the eBird Basic Dataset, a database product where each row of data contains information about time, date and location of observation, and bird species and numbers observed. We downloaded the data on 1st July 2022, and it contained all observations submitted to the dataset prior to that day as it is a real-time platform. To adjust for variations in sample efforts among counties, we used the rarefaction approach to estimate a proxy for bird diversity (i.e., rarefied species richness) [[28], [29], [30]]. Our study utilized bird data collected across the US from 2013 to 2015, which included 6,516,914 events with 104,959,235 observation entries in 3137 counties. On average, each county had 2077 events and 33,458 observation entries. To account for observation bias, we excluded 380 counties with 50 or fewer observation events. After this exclusion, we analyzed data from 2757 counties, where the minimum bird abundance recorded was 907. We used this value as the minimum abundance for the subsample size in the ‘rarefy’ function of the ‘vegan’ package in R language [31,32]. The rarefied species richness derived from this analysis was used in our subsequent data analyses. We used bird data from 2013 to 2015 as it was compatible with the health measurement data from 2014, which was the most recently available health data at the time of our study [33,34]. For sensitivity analysis, we used eBird data of 2019–2021.

### 2.3 Human health data

Population health data, including life expectancy (at birth) and age-specific mortality risk, was obtained from the Institute for Health Metrics and Evaluation (IHME). Research by IHME used small area estimation methods to produce annual life tables and calculate age-specific mortality risks. De-identified death records from the National Center for Health Statistics (NCHS) and population counts from the census bureau, NCHS, and the Human Mortality Database were used in the analysis. This dataset provides estimates for life expectancy and age-specific mortality risk at the county level for each county or county equivalent over 1980–2014 (in every 5 years except for 2014), as well as the percentage change of life expectancy for each region during this period. Data of age-specific mortality risk was provided in the following age categories 0–5, 5–25, 25–45, 45–65, and 65–85 [33].

IHME research applied a novel methodology to death registration data from the National Vital Statistics System in order to estimate annual mortality rates for 21 mutually exclusive causes of death at the county level. This dataset provides estimates for cause-specific age-standardized mortality rates at the county level for each county or county equivalent over 1980–2014 (in every 5 years except for 2014). The 21 mutually exclusive causes of death are listed in elsewhere [34].

### 2.4 Covariates

County-level statistics on population characteristics (i.e., size, gender, age, and ethnicity), socio-economics (i.e., educational level, median household income, gross domestic product per capita, and unemployment and poverty rates), healthcare service (i.e., medical insurance coverage and number of physicians per residential population), and residential environment (i.e., the Rural-Urban Continuum Code) [35], and geographical location (i.e., latitude and longitude) were derived from the US national official sources (Supplementary Table 1). Data from 2014 or in close proximity to 2014 was used. Information on the location of each county was collected using coordinates (latitude and longitude) based on geographic centroids.

### 2.5 Mapping of datasets

**Fig. 1:**
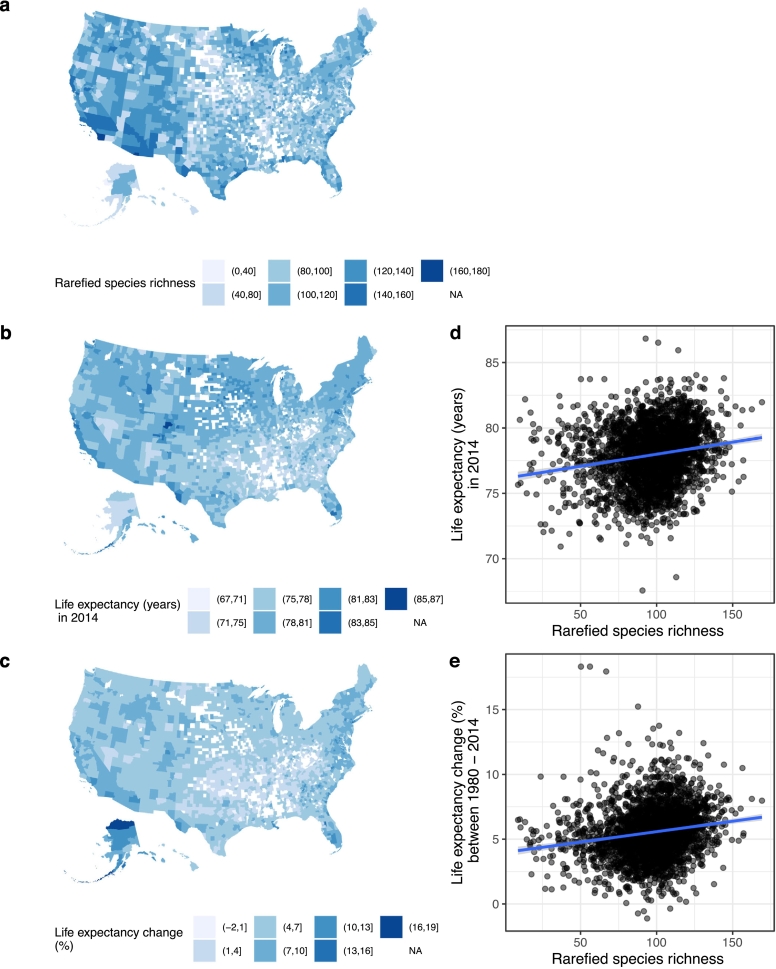
The geographical patterns of rarefied species richness of birds and life expectancy across contiguous US. (a) Rarefied species richness of birds. (b) Life expectancy at birth in 2014. (c) Percentage change of the life expectancy between 1980 and 2014. (d) Scatter plot between the rarefied species richness of birds and life expectancy with a linear prediction line (in blue). (e) Scatter plot between the rarefied species richness of birds and percentage change in life expectancy with a linear prediction line (in blue). (For interpretation of the references to colour in this figure legend, the reader is referred to the web version of this article.)

Databases of rarefied species richness of birds (*n* = 2757), human health and covariates were linked together by county identity, where all variables were complete in 2751 counties (2751/3140, 87·6% of the total US counties). Counties contained missing data of human health or covariate information (*n* = 6) were excluded from analysis. Data imputation for these counties was not attempted as the proportion was very small, and thus 2751 was the total sample size of the studied counties, colored in Fig. 1a-c.

### 2.6 Statistical analysis

Health data in 2014 (i.e., life expectancy at birth, age-specific mortality risk, and cause-specific mortality rate; outcome variables) and data of rarefied species richness of birds (exposure variable) based on 2013–2015 were analyzed. From the longitudinal perspective, the percentage change in life expectancy between 1980 and 2014 was also used as an additional outcome variable measuring health improvement over time.

Descriptive statistics on the studied variables (including rarefied species richness of birds, population characteristics, socio-economics, healthcare services, residential environment and location, and health measurements) were presented at first. Simple statistics (e.g., Pearson's correlation test) was initially carried out to assess the possible correlations between regional rarefied species richness of birds, socio-ecoomic factors, and health measurements.

In regression modelling, univariate linear regression analyses were firstly used for assessing the relationship between individual factors and health measurements, without confounding adjustment. In this stage, we reported the regression coefficient for each assessed factor and the R^2^ for the assessed model, where the former parameter measures the size of effect of each factor per unit on the change of health measurement, and the later parameter reflects the overall magnitude of association. Then, multivariate liner regression analyses were used, which included factors within each domain (biodiversity, population characteristics, socio-economics, healthcare service, residential environment, and geographical location), to assess the independence of associations of the significant factors from the univariate analyses, with adjustment of confounding effects for each other. The regression coefficient was reported for the factors for which a significant association with health measurements remained. Then, all significant factors in the within-domain analyses were included in a final model, with all the factors entered simultaneously at the beginning and a backward selection method was applied to finalize the significant factors after full confounding adjustment.

A *p*-value <0·05, two tailed, was considered statistically significant in all analyses, as this study was exploratory in nature. However, some notes were made for the different levels of significance according to the following thresholds (*p* < 0·05, 0·01 and 0·001) to enable open scrutiny of the results. All the statistical analyses were carried out using STATA (version 15).

## 3 Results

The average rarefied species richness of birds in each studied US county was 97·0 (standard deviation (SD), 22·0). Its geographical pattern is shown in Fig. 1a.

The descriptive statistics of population characteristics, social-economics, healthcare service, residential environment index, and geographical location at county level in the US is presented in Table 1. The average life expectancy of the studied 2751 US counties in 2014 was 77·9 years (SD, 2·2), and the average percentage increase of life expectancy from 1980 to 2014 was 5·5 years (SD, 2·0). Their geographical patterns are shown in Fig. 1b and c. Notably, in the southwestern coast region where the rarefied species richness of birds was relatively high, the life expectancy was also relatively high; whereas in southeastern counties where the rarefied species richness of birds was low, the life expectancy was also low. This suggested a positive correlation, between rarefied species richness of birds and overall health of human population. Scatter plots between regional rarefied species richness of birds and life expectancy at the county level is shown in Fig. 1d (correlation coefficient (r) = 0·18, *p* < 0·001). Correlation was also found with the percentage increase of life expectancy (*r* = 0·17, *p* < 0·001, Fig. 1e).

**Table 1:** County-level statistics of the US.

Variable	Number (%), or median (IQR)
Population information	
Size	31,118 (13,040, 79,614)
Gender, male	49·6% (49·0%, 50·5%)
Ethnicity, white alone	91·9% (81·0%, 95·8%)
Age, years	
0–9	12·1% (10·9%, 13·3%)
10–19	12·9% (11·9%, 13·9%)
20–29	11·8% (10·4%, 13·5%)
30–39	11·5% (10·5%, 12·5%)
40–49	12·2% (11·3%, 13·2%)
50–59	14·7% (13·7%, 15·6%)
60–69	12·0% (10·7%, 13·4%)
70–79	7·2% (6·0%, 8·4%)
80 and over	4·3% (3·5%, 5·2%)
Socio-economic	
Educational level, 25 years and over	
Less than a high school diploma	11·7% (8·6%, 16·3%)
A high school diploma only	33·9% (29·2%, 38·8%)
Completing some college or associate's degree	30·8% (27·5%, 34·3%)
A bachelor's degree or higher	20·0% (15·6%, 26·8%)
Median household income (annual, US dollar)	46,046 (39,999, 53,304)
Unemployment rate, age < 65	6·0% (4·7%, 7·4%)
Poverty rate	15·5% (12·0%, 19·9%)
Gross domestic product per capita (annual, US dollar)	36,880 (27,536, 49,631)
Healthcare service	
Health insurance coverage, age < 65	86·1% (82·4%, 89·7%)
Physicians per 1000 population	0·9 (0·4, 1·6)
Residential environment (Rural-Urban Continuum Code)	
1 (Metro areas, 1 million population or more)	412 (14·9)
2 (Metro areas, 250 thousand to 1 million population)	366 (13·3)
3 (Metro areas, population fewer than 250 thousand)	333 (12·1)
4 (Urban population of 20 thousand or more, adjacent to a metro area)	209 (7·6)
5 (Urban population of 20 thousand or more, not adjacent to a metro area)	89 (3·2)
6 (Urban population of 2500 to 19,999, adjacent to a metro area)	514 (18·7)
7 (Urban population of 2500 to 19,999, not adjacent to a metro area)	361 (13·1)
8 (Completely rural or <2500 urban population, adjacent to a metro area)	166 (6·0)
9 (Completely rural or <2500 urban population, not adjacent to a metro area)	301 (10·9)
Geographic location	
Longitude	−90·3 (−98·6, −83·1)
Latitude	38·5 (34·7, 41·9)

IQR, interquartile range. Based on 2751 studied US counties.

Univariate liner regression analyses initially showed that all the studied exposure variables were associated with life expectancy (Model 1, Table 2). Multivariate regression analyses, conducted within domains, further demonstrated their associations with life expectancy. These domain-focused analyses suggested that the socio-economic domain, which included three significant variables (i.e. education, unemployment and poverty), was the leading contribution domain. This domain explained 56% of the variance in life expectancy, based on the county-level data (Model 2, Table 2). Rarefied species richness of birds, as the only indicator for the biodiversity domain, explained 3% of the variance (Model 2, Table 2). In the final model, higher rarefied species richness of birds remained statistically significant in the association with a longer life expectancy after adjustment for other significant contribution factors (regression coefficient (95% CIs), 0·005 (0·003, 0·008), years of life expectancy per rarefied species richness, see Model 3, Table 2).

**Table 2:** Regression analyses for the county-level relationship between rarefied species richness of birds and life expectancy at birth with adjustment for other potential confounding factors.

Domain Variable	Univariate analyses (Model 1)		Multivariate analyses within domains (Model 2)		Multivariate analysis across all domains (Model 3)	
	Regression coefficient (95% CIs) | R^2^					
Biodiversity				0·033		0·668
Rarefied species richness of birds	0·018 (0·015, 0·022)	0·033	0·018 (0·015, 0·022)		0·005 (0·003, 0·008)	
Population				0·223		
Population size (per 1000,000)	1·099 (0·859, 1·339)	0·029	1·648 (1·429, 1·867)		0·587 (0·425, 0·749)	
Male (%)	0·055 (0·019, 0·092)	0·003	0·063 (0·031, 0·096)		0·119 (0·096, 0·142)	
White alone (%)	0·059 (0·054, 0·064)	0·160	0·065 (0·060, 0·069)		0·030 (0·025, 0·034)	
Age ≥ 70 (%)	0·050 (0·025, 0·076)	0·005	–		–	
Socio-economic				0·562		
A bachelor's degree or higher (%)	0·143 (0·136, 0·150)	0·373	0·091 (0·084, 0·097)		0·116 (0·108, 0·123)	
Median household income (per 1000$)	0·117 (0·111, 0·122)	0·397	–		–	
Unemployment rate, age < 65 (%)	−0·465 (−0·500, −0·430)	0·199	−0·067 (−0·098, −0·035)		–	
Poverty rate (%)	−0·243 (−0·253, −0·233)	0·436	−0·165 (−0·177, −0·153)		−0·107 (−0·120, −0·094)	
GDP per capita (per 1000$)	0·755 (0·163, 1·347)	0·002	–		–	
Healthcare service				0·176		
Health insurance coverage, age < 65 (%)	0·169 (0·154, 0·184)	0·156	0·156 (0·141, 0·171)		–	
Physicians per 1000 population	0·297 (0·248, 0·346)	0·049	0·194 (0·148, 0·241)		−0·063 (−0·101, −0·026)	
Residential environment				0·057		
Rural-Urban Continuum Code						
2 (vs. 1)	−0·762 (−1·069, −0·454)	0·057	−0·762 (−1·069, −0·454)		0·231 (0·038, 0·424)	
3 (vs. 1)	−0·714 (−1·029, −0·398)		−0·714 (−1·029, −0·398)		0·466 (0·259, 0·672)	
4 (vs. 1)	−1·063 (−1·427, −0·699)		−1·063 (−1·427, −0·699)		0·377 (0·140, 0·614)	
5 (vs. 1)	−1.087 (−1·588, −0·586)		−1.087 (−1·588, −0·586)		0·234 (−0·084, 0·551)	
6 (vs. 1)	−1·656 (−1·939, −1·372)		−1·656 (−1·939, −1·372)		0·401 (0·205, 0·611)	
7 (vs. 1)	−1·038 (−1·347, −0·730)		−1·038 (−1·347, −0·730)		0·324 (0·106, 0·541)	
8 (vs. 1)	−1·215 (−1·609, −0·821)		−1·215 (−1·609, −0·821)		0·549 (0·287, 0·810)	
9 (vs. 1)	−0·281 (−0·606, 0·043)		−0·281 (−0·606, 0·043)		0·751 (0·520, 0·982)	
Geographic location				0·190		
Longitude	−0·028 (−0·035, −0·022)	0·028	−0·009 (−0·016, −0·003)		−0·009 (−0·013, −0·005)	
Latitude	0·182 (0·168, 0·196)	0·187	0·175 (0·161, 0·190)		0·059 (0·048, 0·070)	

CIs, confidence intervals. Results were based on 2751 studied US counties.

Results for the percentage change of life expectancy, using the same statistical approach, are shown in Table 3. The analysis suggested that counties where there were higher rarefied species richness of birds were associated with a larger percentage increase in life expectancy over 1980–2014, after consideration of other significant contribution factors (Model 3, Table 3).

**Table 3:** Regression analyses for the county-level relationship between rarefied species richness of birds and percentage change of life expectancy with adjustment for other potential confounding factors.

Domain Variable	Univariate analyses (Model 1)		Multivariate analyses within domains (Model 2)		Multivariate analysis across all domains (Model 3)	
	Regression coefficient (95% CIs) | R^2^					
Biodiversity				0·031		0·396
Rarefied species richness of birds	0·016 (0·013, 0·019)	0·031	0·016 (0·013, 0·019)		0·010 (0·007, 0·012)	
Population				0·099		
Population size (per 1000,000)	1·505 (1·294, 1·712)	0·066	1·421 (1·208, 1·633)		0·405 (0·211, 0·599)	
Male (%)	0·053 (0·020, 0·085)	0·004	0·087 (0·056, 0·119)		0·097 (0·069, 0·124)	
White alone (%)	−0·025 (−0·030, −0·021)	0·037	−0·021 (−0·025, −0·016)		−0·033 (−0·037, −0·029)	
Age ≥ 70 (%)	−0·057 (−0·080, −0·034)	0·008	–		–	
Socio-economic				0·255		
A bachelor's degree or higher (%)	0·101 (0·094, 0·108)	0·230	0·071 (0·062, 0·080)		0·061 (0·052, 0·070)	
Median household income (per 1000$)	0·073 (0·068, 0·079)	0·193	0·036 (0·028, 0·043)		0·039 (0·031, 0·047)	
Unemployment rate, age < 65 (%)	−0·008 (−0·043, 0·028)	0·000	–		–	
Poverty rate (%)	−0·089 (−0·101, −0·077)	0·072	–		–	
GDP per capita (per 1000$)	0·615 (0·082, 1·148)	0·002	–		–	
Healthcare service				0·073		
Health insurance coverage, age < 65 (%)	0·042 (0·028, 0·056)	0·012	0·022 (0·007, 0·036)		−0·056 (−0·071, −0·042)	
Physicians per 1000 population	0·321 (0·277, 0·364)	0·070	0·306 (0·262, 0·351)		–	
Residential environment				0·100		
Rural-Urban Continuum Code						
2 (vs. 1)	−1·212 (−1·483, −0·941)	0·100	−1·212 (−1·483, −0·941)		−0·288 (−0·526, −0·051)	
3 (vs. 1)	−1·314 (−1·592, −1·036)		−1·314 (−1·592, −1·036)		−0·205 (−0·458, 0·048)	
4 (vs. 1)	−1·714 (−2·034, −1·394)		−1·714 (−2·034, −1·394)		−0·332 (−0·625, −0·039)	
5 (vs. 1)	−2·045 (−2·485, −1·604)		−2·045 (−2·485, −1·604)		−0·946 (−1·327, −0·564)	
6 (vs. 1)	−2·032 (−2·281, −1·783)		−2·032 (−2·281, −1·783)		−0·319 (−0·571, −0·068)	
7 (vs. 1)	−1·723 (−1·994, −1·451)		−1·723 (−1·994, −1·451)		−0·451 (−0·720, −0·182)	
8 (vs. 1)	−1·509 (−1·855, −1·162)		−1·509 (−1·855, −1·162)		−0·041 (−0·364, 0·282)	
9 (vs. 1)	−1·212 (−1·498, −0·927)		−1·212 (−1·498, −0·927)		−0·006 (−0·293, 0·281)	
Geographic location				0.056		
Longitude	−0·003 (−0·009, 0·002)	0·000	–		–	
Latitude	0·090 (0·077, 0·104)	0·056	0·090 (0·077, 0·104)		0·094 (0·080, 0·107)	

CIs, confidence intervals. Results were based on 2751 studied US counties.

County-level summary of age-specific mortality risk is shown in Supplementary Table 2. Assessment on age-specific mortality risks showed that the association with bird species richness was significant in the youngest (0–5 years) and older (45–65 and 65–85 years) age categories, with the effect size the largest for the eldest (regression coefficient (95% CIs), −0·015 (−0·021, −0·008), risk of death in % per rarefied species richness, adjusted results, Table 4) due to the highest natural rate of death in the most elderly population.

**Table 4:** Regression analyses for the county-level relationship between rarefied species richness of birds and age-specific mortality risk.

	Rarefied species richness of birds	
Mortality risk	Univariate analyses (unadjusted results): Regression coefficient (95% CIs) | R^2^	Multivariate analyses (adjusted results): Regression coefficient (95% CIs)
Probability of death age 0 to 5	−0·0015 (−0·0018, −0·0011) | 0·0263	−0·0003 (−0·0005, −0·0002)***
Probability of death age 5 to 25	−0·0017 (−0·0021, −0·0012) | 0·0175	−0·0001 (−0·0004, 0·0002), not significant
Probability of death age 25 to 45	−0·0051 (−0·0065, −0·0037) | 0·0175	−0·0007 (−0·0015, 0·0001), not significant
Probability of death age 45 to 65	−0·0239 (−0·0287, −0·0190) | 0·0330	−0·0071 (−0·0099, −0·0043)***
Probability of death age 65 to 85	−0·0497 (−0·0593, −0·0401) | 0·0362	−0·0146 (−0·0212, −0·0080)***

Adjustment for all the studied population characteristics, socio-economic, healthcare service, residential environment, and geographic location variables. Results were based on 2751 studied US counties. ***, association reached the following statistical significance threshold, *p* < 0.001.

County-level summaries of cause-specific mortality rates, including the ranking of cause of death, is shown in Supplementary Table 3. Investigation on the association between rarefied species richness of birds and cause-specific mortality rates is presented in Table 5. After potential confounding adjustment, rarefied species richness of birds was found to be associated with a majority of cause-specific deaths (12 out of 21), including non-communicable diseases (e.g. neoplasms, cardiovascular diseases, and chronic respiratory diseases), and communicable, maternal, neonatal and nutritional diseases. Most associations were in an inverse relationship, indicating its benefit on health in general. The associations with the top causes of deaths were regarded as highly significant, with considerable effect sizes, for example, for cardiovascular diseases (regression coefficient (95% CIs), −0·242 (−0·311, −0·174), death rate in % per rarefied species richness), for neoplasms (−0·101 (−0·139, −0·064)), and for chronic respiratory diseases (−0·059 (−0·082, −0·037)) (adjusted results, Table 5).

**Table 5:** Association of rarefied species richness of birds with rate of cause-specific mortality at the county level.

		Rarefied species richness of birds	
Cause of death		Univariate analyses (unadjusted results): Regression coefficient (95% CIs) | R^2^	Multivariate analyses (adjusted results): Regression coefficient (95% CIs)
Communicable, maternal, neonatal and nutritional diseases			
	HIV/AIDS and tuberculosis	Not significant	–
	Diarrhea, lower respiratory and other common infectious diseases	−0·0885 (−0·1051, −0·0718) | 0·0379	−0·0487 (−0·0623, −0·0351)***
	Neglected tropical diseases and malaria	−0·0003 (−0·0004, −0·0002) | 0·0234	−0·0001 (−0·0002, −0·0001)***
	Maternal disorders	−0·0009 (−0·0012, −0·0007) | 0·0211	−0·0002 (−0·0004, −0·0001)**
	Neonatal disorders	−0·0082 (−0·0102, −0·0061) | 0·0218	−0·0025 (−0·0037, −0·0014)***
	Nutritional deficiencies	−0·0038 (−0·0049, −0·0026) | 0·0145	−0·0010 (−0·0020, −0·0001)*
	Other communicable, maternal, neonatal and nutritional diseases	Not significant	–
Non-communicable diseases			
	Neoplasms	−0·2704 (−0·3206, −0·2202) | 0·0390	−0·1014 (−0·1389, −0·0640)***
	Cardiovascular diseases	−0·5529 (−0·6452, −0·4605) | 0·0477	−0·2423 (−0·3105, −0·1741)***
	Chronic respiratory diseases	−0·1255 (−0·1522, −0·0988) | 0·0299	−0·0593 (−0·0819, −0·0366)***
	Cirrhosis and other chronic liver diseases	0·0177 (0·0055, 0·0299) | 0·0029	0·0336 (0·0234, 0·0438)***
	Digestive diseases	−0·0201 (−0·0240, −0·0161) | 0·0345	−0·0073 (−0·0106, −0·0040)***
	Neurological disorders	Not significant	–
	Mental and substance use disorders	0·0254 (0·0147, 0·0361) | 0·0078	0.0210 (0·0111, 0·0308)***
	Diabetes, urogenital, blood, and endocrine diseases	Not significant	–
	Musculoskeletal disorders	Not significant	–
	Other non-communicable diseases	−0·0118 (−0·0141, −0·0094) | 0·0341	−0·0021 (−0·0034, −0·0007)**
Injuries			
	Transport injuries	−0·0593 (−0·0739, −0·0446) | 0·0224	Not significant
	Unintentional injuries	−0·0292 (−0·0376, −0·0208) | 0·0166	Not significant
	Self-harm and interpersonal violence	Not significant	–
	Forces of nature, war, and legal intervention	Not significant	–

Adjustment for all the studied population characteristics, socio-economic, healthcare, residential environment, and geographic location variables. Results were based on 2751 studied US counties. *, **, and ***, associations reached the following statistical significance threshold, *p* < 0.05, *p* < 0.01 and p < 0.001, respectively.

Sensitivity analysis showed a very strong correlation between the rarefied species richness of birds in 2013–2015 and 2019–2021. For the association with human health data (i.e., life expectancy, age-specific mortality risk, cause-specific mortality rate), analyses, which used bird species data in 2019–2021, demonstrated consistent findings (data not shown).

## 4 Discussion

### 4.1 Biodiversity benefits human health

Our results provided novel evidence that biodiversity may be positively associated with better health. With adjustment of other commonly studied health determinants, the effect of rarefied species richness of birds remained significant to the increased life expectancy across the US. Higher rarefied species richness of birds was significantly associated with reduced mortality risks in both very young and old age groups. The effect was pronounced enough to be evident in a significant proportion and type of cause-specific death, including premature mortality due to those top medical problems such as cardiovascular diseases, neoplasms, and chronic respiratory diseases. To the knowledge of the authors, no prior research has reported any similar research. In the literature, most previous research on biodiversity and human health/well-being focused on the mental aspects. A (European-continent) large-scale research in ecological economics demonstrated that richness of bird species was positively associated with life-satisfaction across Europe, and in terms of magnitude it was suggested that the correlation between bird diversity and life-satisfaction was similar to that of income [22]. An epidemiological study across German counties showed plant and bird species richness were positively related to the mental components of the Short Form-12 which is a self-reported scoring system for quality of life, whereas the study did not find significant association with the physical components [4]. However, the relationship is not consistent across all studies; a small-scale research in the setting of a neotropical city suggested that bird diversity were not related to psychological well-being of urban residents [36]. Whilst our method does not establish a causal relationship between bird diversity and health outcomes, there is a plausible mechanism suggesting that this association is meaningful. For example, Marselle and colleagues presented a comprehensive framework of pathways through which both exposure to and experience of biodiversity can be linked to human health [18]. The four domains including positive interactions such as facilitating stress recovery, encouraging physical activity, as well as negative interactions such as increasing risk of allergies and pathogens. Our large-scale ecological study, which utilized county-level data from the US, unveiled a cumulative positive effect arising from the complex interactions between biodiversity and health. Compared to socio-economic factors in the univariate analysis, we found that one unit higher of rarefied species richness of birds was associated with a prolonged life expectancy equivalent to the effect of approximately 150 USD higher annual household income (Model 1, Table 2).

### 4.2 Bird richness as appropriate biodiversity indicator

A variety of taxa have been used as biodiversity indicators in health–environment investigations, including herbaceous plants, trees, insects, birds, small mammals [14,22,37]. Greenspace is also a commonly used biodiversity surrogate [38,39]. In our nationwide study, bird species richness has shown to be a good biodiversity indicator for a few reasons. First, there is a high spatial disparity of bird richness across the study region [40], which results in a clear pattern of biodiversity–health relationships to emerge at the county-level. Second, there is a high availability of citizen science bird monitoring programs in the US and also globally [25,41]. Third, indicators such as greenspaces, trees, or herbaceous plants are more likely to be correlated with socio-economic factors. In urban areas, for instance, socio-economic advantaged communities usually are associated with higher proportions of greenspaces. However, the richness of bird serves as a biodiversity variable relatively independent from human influence, especially when the study takes place at the regional scale [40]. Therefore, our study highlights the effectiveness and cost-efficiency of using bird species richness as a biodiversity indicator to understand large-scale relationships between health and the environment.

### 4.3 Implication for One Health and biodiversity conservation

Our results suggested that biodiversity can reduce the risk of many cause-specific premature deaths. It supported the holistic viewpoint of the One Health approach, emphasizing the interlinkages between human health, the health of the shared environment, and the well-being of organisms therein [1,12]. In the Anthropocene, emerging societal challenges of biodiversity loss, climate change, environment pollution, ecosystem degradation, food security and human health are intertwined [42]. The COVID-19 pandemic has triggered the propagation of ill-considered messages to the public, framing nature as a threat to health [43]. Indeed, >75% of zoonoses that emerged since the 1970s originated from wildlife [44,45]. Yet the same period also witnessed an unprecedented large-scale land cover and land use change, resulting in intensified loss and fragmentation of wildlife habitats [46]. However, our analysis identified a significant negative association between rarefied species richness of birds and mortality rates caused by diarrhea, lower respiratory, and other common infectious diseases (as shown in Table 5). The findings of our study suggest that efforts to mitigate the threats to biodiversity can have a positive impact on human health, and vice versa. The overall positive correlation between biodiversity and human health has a far-reaching implication for fostering synergies between UN's Sustainable Development Goals related to human health (e.g., SDG 3) and nature protection (e.g., SDG 15).

However, it should be noted that biodiversity's beneficial impacts may not be evenly distributed among social groups. For example, the Luxury Effect proposes that higher income levels can promote biodiversity [47,48]. Although our study detected no significant correlation between county-level GDP and rarefied species richness of birds, we revealed a significant positive association between bird diversity with deaths caused by mental and substance use disorders (Table 5). This pattern may be attributed to the disproportionately high mortality rates reported in the southwestern states with Native American reservations [34], which also happen to be the most bird species-rich region in our study (Fig. 1a). Therefore, further studies are necessary to understand the relationship between health and biodiversity in specific social, cultural and population contexts.

The Kunming-Montreal Global Biodiversity Framework marks a paradigm shift in nature conservation, which aims to promote transformative change of conventional nature conservation ideologies and strategies, including the ones that solely emphasize intrinsic values of nature, wilderness, and separate nature from human societies [17]. A new conservation paradigm is in its inception phrase, which also acknowledges relational and instrumental values of nature. This includes values based on people's interactions with nature, as well as the positive contributions that nature provides to people [49,50]. To maximize benefits of biodiversity to human health, more efforts should be made to illustrate the pathways and socio-ecological mechanisms underlying biodiversity–health interactions. This knowledge will inform the implementation of One Health, promoting healthy human–nature interactions in a variety of landscapes from protected areas to urban greenspaces. Conservation biologists, public health experts and landscape planners should work together to design, build and manage the shared environment in novel ways so that human's encounters to nature will benefit both people's mental and physical health, and the health benefits of biodiversity can be shared equitably among social groups [[51], [52], [53]].

### 4.4 Strength and limitation of study

The availability and quality of bird diversity and health data across the US offers a novel opportunity (i.e. linked database research) to investigate environmental epidemiology questions at a very large geographical scale. Ecological study, using data reported at the county level, is a feasible and effortful approach, as in the US counties are the smallest administrative units where essential studied information can be provided [33]. Access to a vast database facilitated this study to have a sufficient sample size and wide geographical coverage of the US, enhancing the generalizability of the results. This study also benefited from the US being a well-developed country with relatively good healthcare budgets and systems across the whole nation, compared to the developing world where regional difference (e.g., capital city vs. urban area vs. rural area) may contribute more significantly to the quality of healthcare and therefore the life span. Nevertheless, in the current analysis, we made great efforts to collect and adjusted for many potential confounding factors at the following domains: population characteristics, socio-economics, healthcare service, residential environment and geographic location, in order to minimize the confounding effects. However, other variables (such as climate and weather details, air conditions, and landscape parameters), which differ among counties, were not available, which became one obvious limitation of this study. Indicators of overall population health such as life expectancy and mortality, were used in this study, instead of more specific measurements such as status of certain diseases and mental conditions. It should be noted that there can be non-negligible variations among the standards of disease identification and recording across the US, and unbiased information of specific health measurements is scarce. Furthermore, many health indicators would be affected by detection capability and suffered from survival bias. For example, a record of higher prevalence of lung cancer in one region could be actually attributed to a better cancer diagnosis method, or a better treatment which results in a longer survival period. However, life expectancy and mortality data (from the national death registration database with information knowing the cause of death) are less problematic particularly in geographically large-scale research across multiple administration areas, and they provide a less biased evaluation on overall population health. As an ecological study in design, this study might also suffer possible biases such as migration bias. We were unable to rule out potential reporting bias across different counties, while the related data has been validated and released from official sources. In addition, we were unable to differentiate between the impacts of biodiversity on deaths caused by mental disorders and those caused by substance use disorders, as both factors fall within a single category. Regarding the data of bird species richness, eBird suggested the following items that may be associated with potential biases: species detectability according to specific birds, variety of users with different skills, issues with reporting exact geographic location, and an uneven distribution of the birding community [25]. This epidemiological study has made no attempt to reveal possible mechanisms behind the observed associations. Future work, such as longitudinal and experiential studies, is necessary to examine the pathways and mediators related to this topic. This study focused on life expectancy and cause-specific mortality, rather than other important indicators of quality of life and well-being that are of interest to One Health researchers. Future work should explore whether the relationships between bird species richness and other quality of life and well-being indicators exhibit similar patterns.

## Authors' contributions

LL and YC conceptualized the study. YC, PZ, QX, DL and BQ analyzed the data. YC and LL drafted the initial manuscript. SC provided substantial comments on the discussion of results and the writing of the manuscript. All authors contributed to the study design and interpretation of the data. All authors approved the final version of the manuscript submitted for publication.

## Declaration of Competing Interest

None.

## Data Availability

The data supporting the findings of this study are openly available (see the Methods section for details).

## Appendix A Supplementary data

Supplementary material Image 1

## References

[1] UNEP (2017).

[2] CBD (2021).

[3] Lindley S.J., Cook P.A., Dennis M., Gilchrist A., Marselle M.R., Stadler J., Korn H., Irvine K.N., Bonn A. (2019). Biodiversity and Health in the Face of Climate Change.

[4] Methorst J., Bonn A., Marselle M., Böhning-Gaese K., Rehdanz K. (2021). Species richness is positively related to mental health – a study for Germany. Landsc. Urban Plan..

[5] Dallimer M., Irvine K.N., Skinner A.M.J., Davies Z.G., Rouquette J.R., Maltby L.L., Warren P.H., Armsworth P.R., Gaston K.J. (2012). Biodiversity and the feel-good factor: understanding associations between self-reported human well-being and species richness. BioScience..

[6] Antonovsky A. (1979). New Perspectives on Mental and Physical Well-Being.

[7] Lindström B., Eriksson M. (2005). Salutogenesis. J. Epidemiol. Community Health.

[8] Schwabe C.W. (1964).

[9] Lee K., Brumme Z.L. (2012). Operationalizing the one health approach: the global governance challenges. Health Policy Plan..

[10] Das Neves C.G. (2020).

[11] Zinsstag J., Schelling E., Crump L., Whittaker M., Tanner M., Stephen C. (2020).

[12] WHO, Connecting global priorities: biodiversity and human health (2015).

[13] Viegas C., Moniz G., Pargana J., Marques S., Resende C., Martins C., Arez A.P., Ceratto N., Viegas S. (2021).

[14] Civitello D.J., Cohen J., Fatima H., Halstead N.T., Liriano J., McMahon T.A., Ortega C.N., Sauer E.L., Sehgal T., Young S. (2015). Biodiversity inhibits parasites: broad evidence for the dilution effect. Proc. Natl. Acad. Sci..

[15] Hough R.L. (2014). Biodiversity and human health: evidence for causality?. Biodivers. Conserv..

[16] Sandifer P.A., Sutton-Grier A.E., Ward B.P. (2015). Exploring connections among nature, biodiversity, ecosystem services, and human health and well-being: opportunities to enhance health and biodiversity conservation. Ecosyst. Serv..

[17] IPBES (2021).

[18] Marselle M.R., Hartig T., Cox D.T., de Bell S., Knapp S., Lindley S., Triguero-Mas M., Böhning-Gaese K., Braubach M., Cook P.A. (2021). Pathways linking biodiversity to human health: a conceptual framework. Environ. Int..

[19] IPBES (2021). Scoping Report for a Thematic Assessment of the Interlinkages among Biodiversity, Water, Food, and Health. https://ipbes.net/sites/default/files/2021-07/20210719_scoping_report_for_the_nexus_assessment.pdf.

[20] Fraixedas S., Lindén A., Piha M., Cabeza M., Gregory R., Lehikoinen A. (2020). A state-of-the-art review on birds as indicators of biodiversity: advances, challenges, and future directions. Ecol. Indic..

[21] Gill F.B., Prum R.O., Robinson S. (2019). https://book.douban.com/subject/30421751/.

[22] Methorst J., Rehdanz K., Mueller T., Hansjürgens B., Bonn A., Böhning-Gaese K. (2021). The importance of species diversity for human well-being in Europe. Ecol. Econ..

[23] Cornell Lab of Ornithology (2021). eBird, EBird. https://ebird.org/home.

[24] Wood C., Sullivan B., Iliff M., Fink D., Kelling S. (2011). eBird: engaging birders in science and conservation. PLoS Biol..

[25] Sullivan B.L., Wood C.L., Iliff M.J., Bonney R.E., Fink D., Kelling S. (2009). eBird: a citizen-based bird observation network in the biological sciences. Biol. Conserv..

[26] Ferreira N., Lins L., Fink D., Kelling S., Wood C., Freire J., Silva C. (2011). BirdVis: visualizing and understanding bird populations. IEEE Trans. Vis. Comput. Graph..

[27] Feng M.-L.E., Che-Castaldo J. (2021). Comparing the reliability of relative bird abundance indices from standardized surveys and community science data at finer resolutions. PLoS One.

[28] Heck K.L., van Belle G., Simberloff D. (1975). Explicit calculation of the rarefaction diversity measurement and the determination of sufficient sample size. Ecology..

[29] Hurlbert S.H. (1971). The nonconcept of species Diversity: a critique and alternative parameters. Ecology..

[30] McMurdie P.J., Holmes S. (2014). Waste not, want not: why rarefying microbiome data is inadmissible. PLoS Comput. Biol..

[31] R Core Team, R: A Language and Environment for Statistical Computing, (n.d.). https://www.r-project.org/ (accessed January 29, 2023).

[32] Oksanen J., Simpson G.L., Blanchet F.G., Kindt R., Legendre P., Minchin P.R., O'Hara R.B., Solymos P., Stevens M.H.H., Szoecs E., Wagner H., Barbour M., Bedward M., Bolker B., Borcard D., Carvalho G., Chirico M., Caceres M.D., Durand S., Evangelista H.B.A., FitzJohn R., Friendly M., Furneaux B., Hannigan G., Hill M.O., Lahti L., McGlinn D., Ouellette M.-H., Cunha E.R., Smith T., Stier A., Braak C.J.F.T. (2017). J. Weedon, vegan: Community Ecology Package. https://CRAN.R-project.org/package=vegan.

[33] Dwyer-Lindgren L., Bertozzi-Villa A., Stubbs R.W., Morozoff C., Mackenbach J.P., van Lenthe F.J., Mokdad A.H., Murray C.J.L. (2017). Inequalities in life expectancy among US counties, 1980 to 2014: temporal trends and key drivers. JAMA Intern. Med..

[34] Dwyer-Lindgren L., Bertozzi-Villa A., Stubbs R.W., Morozoff C., Kutz M.J., Huynh C., Barber R.M., Shackelford K.A., Mackenbach J.P., van Lenthe F.J., Flaxman A.D., Naghavi M., Mokdad A.H., Murray C.J.L. (2016). US County-level trends in mortality rates for Major causes of death, 1980-2014. JAMA..

[35] USDA (2021). ERS - Rural-Urban Continuum Codes. https://www.ers.usda.gov/data-products/rural-urban-continuum-codes.aspx.

[36] Fisher J.C., Bicknell J.E., Irvine K.N., Hayes W.M., Fernandes D., Mistry J., Davies Z.G. (2021). Bird diversity and psychological wellbeing: a comparison of green and coastal blue space in a neotropical city. Sci. Total Environ..

[37] Hassell J.M., Bettridge J.M., Ward M.J., Ogendo A., Imboma T., Muloi D., Fava F., Robinson T.P., Begon M., Fèvre E.M. (2021). Socio-ecological drivers of vertebrate biodiversity and human-animal interfaces across an urban landscape. Glob. Chang. Biol..

[38] Nilon C.H., Aronson M.F., Cilliers S.S., Dobbs C., Frazee L.J., Goddard M.A., O’Neill K.M., Roberts D., Stander E.K., Werner P. (2017). Planning for the future of urban biodiversity: a global review of city-scale initiatives. Bioscience..

[39] Aerts R., Honnay O., Van Nieuwenhuyse A. (2018). Biodiversity and human health: mechanisms and evidence of the positive health effects of diversity in nature and green spaces. Br. Med. Bull..

[40] O’Connor R.J., Jones M.T., White D., Hunsaker C., Loveland T.O.M., Jones B., Preston E. (1996). Spatial partitioning of environmental correlates of avian biodiversity in the conterminous United States. Biodivers. Lett..

[41] Callaghan C.T., Martin J.M., Major R.E., Kingsford R.T. (2018). Avian monitoring–comparing structured and unstructured citizen science. Wildl. Res..

[42] CBD (2021).

[43] Gregg E.A., Kusmanoff A.M., Garrard G.E., Kidd L.R., Bekessy S.A. (2021). Biodiversity conservation cannot afford COVID-19 communication bungles. Trends Ecol. Evol..

[44] WHO (2007).

[45] Jones K.E., Patel N.G., Levy M.A., Storeygard A., Balk D., Gittleman J.L., Daszak P. (2008). Global trends in emerging infectious diseases. Nature..

[46] Secretariat of the Convention on Biological Diversity (2014).

[47] Hope D., Gries C., Zhu W., Fagan W.F., Redman C.L., Grimm N.B., Nelson A.L., Martin C., Kinzig A., Marzluff J.M., Shulenberger E., Endlicher W., Alberti M., Bradley G., Ryan C., Simon U., ZumBrunnen C. (2008). Urban Ecology.

[48] Chamberlain D.E., Henry D.A.W., Reynolds C., Caprio E., Amar A. (2019). The relationship between wealth and biodiversity: a test of the luxury effect on bird species richness in the developing world. Glob. Chang. Biol..

[49] Díaz S., Pascual U., Stenseke M., Martín-López B., Watson R.T., Molnár Z., Hill R., Chan K.M., Baste I.A., Brauman K.A. (2018). Assessing nature’s contributions to people. Science..

[50] Schröter M., Başak E., Christie M., Church A., Keune H., Osipova E., Oteros-Rozas E., Sievers-Glotzbach S., van Oudenhoven A.P.E., Balvanera P., González D., Jacobs S., Molnár Z., Pascual U., Martín-López B. (2020). Indicators for relational values of nature’s contributions to good quality of life: the IPBES approach for Europe and Central Asia. Ecosyst. People..

[51] Heiland S., Weidenweber J., Ward Thompson C. (2019). Biodiversity and Health in the Face of Climate Change.

[52] Kuras E.R., Warren P.S., Zinda J.A., Aronson M.F., Cilliers S., Goddard M.A., Nilon C.H., Winkler R. (2020). Urban socioeconomic inequality and biodiversity often converge, but not always: a global meta-analysis. Landsc. Urban Plan..

[53] Murugesan V. (2020). How can urban planners address emerging zoonoses? A scoping review of recommended interventions. Urbana..

