# Supplementary material for: Relating biodiversity with health disparities of human population: An ecological study across the United States
Source: One Health. 2023 Apr 23;16:100548. doi: 10.1016/j.onehlt.2023.100548 (PMC10288075; doi:10.1016/j.onehlt.2023.100548)
Supplement: Supplementary file 1 — Supplementary material [file mmc1.docx]

**Supplementary Table 1. Data sources of population statistics at the US county-level.**

| Variable | Data file | Data source |
| --- | --- | --- |
| Population characteristics (including size, gender, age and ethnic distribution) | County Characteristics Resident Population Estimates, 2014 data | US Census Bureau |
| Gross domestic product per capita | BEA RGDP per Capita Data for Digest, 2014 data | US Bureau of Economic Analysis |
| Adult education level | American Community Survey 5-year Average County-level Estimates, data of 2014-18 | US Census Bureau |
| Annual median household income | Small Area Income and Poverty Estimates Program, 2014 data | US Census Bureau |
| Unemployment rate | Local Area Unemployment Statistics, 2014 data | US Census Bureau |
| Poverty rate | Small Area Income and Poverty Estimates Program, 2014 data | US Census Bureau |
| Health insurance coverage | Small Area Health Insurance Estimates Program, 2014 data | US Census Bureau |
| Number of physicians per residential population | Area Health Resources, 2014 data | Health Resources and Services Administration |
| Residential environment (rural vs. urban) | The latest version of Rural-Urban Continuum Code, 2013 | US Department of Agriculture Economic Research Service of |

**Supplementary Table 2. County-level summary of age-specific mortality risks**

|  | | Probability of death, (%) | | | | |
| --- | --- | --- | --- | --- | --- | --- |
| Age range | | Median IQR | | | Mean SD | |
|  | 0 to 5 years | 0·66 | 0·55 | 0·80 | 0·69 | 0·20 |
|  | 5 to 25 years | 0·90 | 0·74 | 1·10 | 0·94 | 0·28 |
|  | 25 to 45 years | 2·89 | 2·41 | 3·50 | 3·01 | 0·84 |
|  | 45 to 65 years | 12·58 | 10·87 | 14·81 | 12·97 | 2·89 |
|  | 65 to 85 years | 52·01 | 48·17 | 55·80 | 51·91 | 5·74 |

IQR, interquartile range. SD, standard deviation. Results were based on 2,751 studied US counties.

**Supplementary Table 3. County-level summary of cause-specific mortality rates**

|  | | Number of deaths per 100,000 population | | | | | |
| --- | --- | --- | --- | --- | --- | --- | --- |
| Cause of Death | | Median IQR | | | Mean SD | | Rank |
| Communicable, maternal, neonatal and nutritional diseases | |  |  |  |  |  |  |
|  | HIV/AIDS and tuberculosis | 0·93 | 0·57 | 1·71 | 1·55 | 2·07 | (17) |
|  | Diarrhea, lower respiratory and other common infectious diseases | 31·48 | 25·91 | 38·25 | 32·62 | 9·99 | (6) |
|  | Neglected tropical diseases and malaria | 0·05 | 0·04 | 0·09 | 0·07 | 0·05 | (21) |
|  | Maternal disorders | 0·32 | 0·26 | 0·41 | 0·35 | 0·14 | (19) |
|  | Neonatal disorders | 3·02 | 2·47 | 3·79 | 3·30 | 1·21 | (14) |
|  | Nutritional deficiencies | 1·49 | 1·10 | 1·91 | 1·58 | 0·68 | (16) |
|  | Other communicable, maternal, neonatal and nutritional diseases | 1·20 | 1·03 | 1·43 | 1·25 | 0·32 | (18) |
| Non-communicable diseases | |  |  |  |  |  |  |
|  | Neoplasms | 202·91 | 184·31 | 223·19 | 202·91 | 30·10 | (2) |
|  | Cardiovascular diseases | 267·77 | 232·88 | 308·62 | 273·22 | 55·61 | (1) |
|  | Chronic respiratory diseases | 61·75 | 51·56 | 72·45 | 62·74 | 15·94 | (4) |
|  | Cirrhosis and other chronic liver diseases | 17·30 | 13·96 | 20·89 | 18·32 | 7·18 | (10) |
|  | Digestive diseases | 15·96 | 14·50 | 17·46 | 15·99 | 2·37 | (11) |
|  | Neurological disorders | 96·40 | 81·41 | 111·42 | 96·42 | 21·71 | (3) |
|  | Mental and substance use disorders | 11·83 | 8·72 | 16·02 | 13·11 | 6·31 | (12) |
|  | Diabetes, urogenital, blood, and endocrine diseases | 60·17 | 50·72 | 70·92 | 60·17 | 16·96 | (5) |
|  | Musculoskeletal disorders | 3·10 | 2·70 | 3·63 | 3·23 | 0·78 | (15) |
|  | Other non-communicable diseases | 6·52 | 5·68 | 7·42 | 6·66 | 1·40 | (13) |
| Injuries | |  |  |  |  |  |  |
|  | Transport injuries | 21·23 | 15·66 | 27·36 | 22·04 | 8·71 | (8) |
|  | Unintentional injuries | 23·40 | 20·53 | 26·20 | 23·62 | 4·98 | (7) |
|  | Self-harm and interpersonal violence | 20·83 | 17·07 | 24·93 | 21·72 | 6·87 | (9) |
|  | Forces of nature, war, and legal intervention | 0·07 | 0·04 | 0·10 | 0·08 | 0·09 | (20) |

IQR, interquartile range. SD, standard deviation. Rank, based on the mean value, starting from the most frequent cause of death. Results were based on 2,751 studied US counties.
